# Translation and Validation of the Basic Psychological Need Satisfaction in Active Commuting to and from School (BPNS-ACS) Scale in Young Portuguese Students

**DOI:** 10.3390/ijerph182413091

**Published:** 2021-12-11

**Authors:** Adilson Marques, Thiago Santos, Élvio R. Gouveia, Yolanda Demetriou, Dorothea M. I. Schönbach, Gerson Ferrari, Dorota Kleszczewska, Anna Dzielska, Miguel Peralta

**Affiliations:** 1 Centro Interdisciplinar de Performance Humana (CIPER), Faculdade de Motricidade Humana, Universidade de Lisboa, 1499-002 Lisboa, Portugal; mperalta@fmh.ulisboa.pt; 2 Instituto de Saúde Ambiental (ISAMB), Faculdade de Medicina, Universidade de Lisboa, 1649-028 Lisboa, Portugal; 3 Faculdade de Ciências Sociais e Tecnologia, Universidade Europeia, 1500-210 Lisboa, Portugal; thiago_os@hotmail.com; 4 Departamento de Educação Física e Desporto, Universidade da Madeira, 9000-390 Funchal, Portugal; erubiog@staff.uma.pt; 5 Interactive Technologies Institute, LARSyS, 9020-105 Funchal, Portugal; 6 Department of Sport and Health Sciences, Technical University of Munich, 80992 Munich, Germany; yolanda.demetriou@tum.de (Y.D.); dorothea.schoenbach@tum.de (D.M.I.S.); 7 Escuela de Ciencias de la Actividad Física, el Deporte y la Salud, Universidad de Santiago de Chile (USACH), Santiago 7500618, Chile; gerson.demoraes@usach.cl; 8 Institute of Mother and Child Foundation, 01-211 Warsaw, Poland; dorota.kleszczewska@imid.med.pl; 9 Department of Child and Adolescent Health, Institute of Mother and Child, 01-211 Warsaw, Poland; anna.dzielska@imid.med.pl

**Keywords:** school, walking, cycling, active transportation

## Abstract

Active commuting to and from school (ACS) is a strategy to enhance physical activity levels in youths. To promote ACS, it is important to understand the factors that lead to this behaviour. With this in mind, an adaptation of the Basic Psychological Needs in Exercise Scale for ACS was developed, named the Basic Psychological Need Satisfaction in Active Commuting to and from School (BPNS-ACS) scale. This study aimed to translate and evaluate the psychometric properties of the BPNS-ACS scale in young Portuguese students. A cross-sectional study was designed. A total of 338 students (212 girls, 126 boys), aged between 11 and 19 years old from 31 Portuguese cities participated in this study. To provide validity evidence based on the questionnaire’s internal structure, confirmatory factor analyses were performed to test the three dimensions of the BNPS-ACS scale. The results of the confirmatory factor analysis indicated an acceptable fit to the data. The internal consistency of the measures was accepted as the composite reliability values ranged from 0.78 to 0.94. The evaluation of psychometric properties provided evidence of the adequacy of this questionnaire among Portuguese youth aged 11 to 19 years old.

## 1. Introduction

The health benefits of physical activity for youth are well-known [1]. As a result, youth worldwide are recommended to engage in at least 60 min of daily moderate-to-vigorous physical activity to benefit their health [2]. However, it is estimated that most young people (around 80%) do not fulfil this recommendation [3,4]. The low global prevalence of physical activity is worrying, and urgent action is needed to promote this health behaviour.

Given this picture, public health authorities have been promoting active commuting as an effective strategy to enhance physical activity levels, especially active commuting to and from school (ACS), among young people. This strategy is supported by several investigations showing that ACS is associated with greater physical activity levels in both boys and girls [5,6,7] and can contribute to a significant amount of youth’s daily physical activity [8]. Notwithstanding, to promote physical activity through ACS, it is important to understand the main factors that lead to this behaviour in young people [9].

The self-determination theory (SDT) [10,11] provides a conceptual framework to approach human behaviour, which is widely used in the context of physical activity in young people [12]. Within the SDT framework, the basic psychological needs (BPN) theory proposes autonomy, competence, and relatedness as the three essential, innate, and universal needs that regulate and impel motivation and functioning of natural behavioural propensities [10,13,14]. According to the BPN theory, the satisfaction of these needs (autonomy, competence, and relatedness), driven by supportive environments and significant social agents (e.g., relatives, peers), leads to autonomous forms of motivation [10,15]; on the other hand, unfulfilling BPN driven by unsupportive environments providing significant social agents is associated with controlled forms of motivation [10,15]. Therefore, the fulfilment of the BPN or the failure in doing so are related to positive cognitive, affective, and behavioural outcomes or negative and maladaptive outcomes, respectively.

Autonomy, competence, and relatedness need satisfaction has been previously associated with several health-related outcomes in multiple life domains, including physical activity [16,17,18,19,20]. However, it has not been thoroughly examined in the ACS setting [21]. Examining whether young people’s BPN satisfaction is related with ACS would be helpful for better understanding this behaviour. For this, having a specific questionnaire for ACS is of particular importance, as its context is different from that of engaging in physical activity during leisure time. Due to this, an adaptation of the Basic Psychological Needs in Exercise Scale for ACS was developed by Burgueño, González-Cutre, Sevil-Serrano, Herrador-Colmenero, Segura-Díaz, Medina-Casaubón and Chillon [21], named the Basic Psychological Need Satisfaction in Active Commuting to and from School (BPNS-ACS) scale. This instrument consists of 12 items grouped into four items per factor to measure BPN satisfaction including autonomy, competence, and relatedness. As suggested by the SDT [10,14], autonomy relates to the need to feel ownership of one’s behaviour, competence is associated with the need to produce desired outcomes and to experience mastery, and relatedness refers to the need to feel connected to others. The BPNS-ACS scale was shown to be a valid and reliable questionnaire to measure the three BPN satisfaction in ACS among Spanish youth [21]. Furthermore, the used of a three-factor confirmatory factor approach revealed better fit indexes for the 12-item grouped in autonomy, competence, and relatedness than other approaches, and were show to remain invariant across gender and age [21].

To investigate BPN satisfaction of physical activity behaviour in ACS and to better understand this behaviour in young people from other native languages than Spanish, there is the need to translate and validate this questionnaire in other languages. Therefore, this study aimed to translate and evaluate the psychometric properties of the BPNS-ACS scale in young Portuguese students.

## 2. Materials and Methods

### 2.1. Participants

A total of 338 students (212 girls and 126 boys) aged between 11 and 19 years old (mean age (standard deviation) = 15.56 (2.08)) from 31 Portuguese cities (continental and Madeira island) participated in this study. Altogether, 166 students were from middle schools (5th to 9th grades) and 207 from high schools (10th to 12th grades).

Overall, 63.0% of students had their bikes, and 83.8% knew how to ride them. In addition, 91.9% of students reported that close family had a car. Most students reported usually using a passive mode (42.2% by car, 37.9% public transportation, and 0.3% scooter) when commuting to and from school, compared to fewer students reporting an active mode (17.9% walking and 0.3% cycling). Simultaneously, we observed that 68.5% of students did not walk to and from school on a single day, while 5.2% walked on one day per week, 4% on two days per week, 2.0% on three days per week, 2.6% on four days per week and 17.6% walked every day. Regarding cycling, the great majority (98.8%) of students never used the bike to commute to school or back home.

### 2.2. Questionnaire

The BPNS-ACS scale was previously validated in a sample of Spanish young people [21]. This questionnaire consists of 12 items grouped into four items per factor measuring: autonomy, competence, and relatedness need satisfaction. The questionnaire begins with the following statement: “What do you think about your usual mode of commuting to and from school?” The response for each item was collected using a five-point Likert scale, from 1 (strongly disagree) to 5 (strongly agree).

ACS behaviour was assessed by asking for the usual mode of commuting to and from school. The following response options were possible, only selecting one: walking, cycling, by car, by motorcycle, by bus, or other (in this case, reporting the mode was required). Similar to previous research, students who reported commuting by walking or cycling to and from school were categorised as active commuters. Students who reported commuting to and from school by car, motorcycle, or bus were categorised as passive commuters [22].

### 2.3. Procedures

A cross-sectional study was designed, developed in two main stages: (1) translation and cultural adaptation of the questionnaire; (2) evaluation of the psychometric properties. All students included in the study participated voluntarily and were informed about the study aims, procedures and characteristics. Beforehand, informed consent to participate in the study was obtained from the students’ legal guardians.

#### 2.3.1. Translation and Cultural Adaptation to Portuguese

The Spanish BPNS-ACS scale was translated into Portuguese by two independent Portuguese native translators, which resulted in two Portuguese versions of the questionnaire. The translators compared and discussed the two versions and reached a consensus to obtain the first Portuguese consensual version. Then, a committee of three expert researchers in this field evaluated the semantic, idiomatic, conceptual and cultural equivalences of the questionnaire. These expert researchers provided recommendations on the intelligibility of the questionnaire’s instructions and questions. This process resulted in the Portuguese consensual version 2. Afterwards, the Portuguese consensual version 2 was sent for back-translation to two other independent bilingual translators. This step aimed to assess whether the Portuguese version reflected the content of the original version in Spanish. This stage generated two back-translations, which were analysed together with the translators and expert researchers. Lastly, the Portuguese consensual version 2 was submitted to a small sample of 6 students (3 boys and 3 girls) to test the questionnaires’ acceptability, understanding and adequacy. Students’ feedback was taken into account, resulting in the final Portuguese version of the BPNS-ACS scale.

#### 2.3.2. Evaluation of the Psychometric Properties

To evaluate the psychometric properties and validity of the Portuguese version of the BPNS-ACS scale, the questionnaire was applied to a sample of 338 students between June and August 2021. Students fulfilled the questionnaire online through a link sent to their legal guardians after obtaining the informed consent. Students were encouraged to sincerely answer the questionnaire with their perception, as there were no right or wrong answers.

### 2.4. Statistical Analysis

Data were analysed using AMOS 26.0 (SPSS Inc, Chicago, IL, USA). Given that an “a prior” theoretical structure of the basic need satisfaction scale was proposed, there were no requirements for performing an exploratory factor analysis [23]. To provide validity evidence based on the questionnaire’s internal structure, confirmatory factor analyses were performed to test the three dimensions of the BNPS-ACS scale (i.e., autonomy, competence, and relatedness need satisfaction) proposed by Burgueño and colleagues (2020). The maximum likelihood method with the bootstrapping procedure was selected given the lack of multivariate normality (Mardia’s coefficient = 48.99, *p* < 0.01) [24]. This bootstrapping procedure solves the violation of multivariate normality assumption through 5000 random replication samples based on the original sample [24]. This enables us to estimate the standard error and the confidence interval at 95% (95% CI) for each statistical parameter.

A good fit of the model was assumed when the ratio of the chi-square (χ^2^) to its degrees of freedom (df) was less than or equal to 3.0 (Hair et al., 2018), the Comparative-of-Fit-Index (CFI), the Incremental Fit Index (IFI), the Tucker–Lewis Index (TLI) were larger than or equal to 0.90 [25]. The Root Mean Square Error of Approximation (RMSEA) with its confidence interval at 90% (90% CI) and the Standardised Root Mean Square Residual (SRMR) value was below the minimum or approximated to the cut-off point of 0.07 [26]. The standardised regression weights were acceptable with values above 0.50 [25]. Convergent validity was evaluated through the Average Variance Extracted (AVE), and values equal to or greater than 0.50 were considered indicative of good convergent validity [27]. Additionally, discriminant validity was accepted when the AVE of each construct was greater than the squared multiple correlations between that construct and any other [27].

Finally, a multi-group factor analysis of invariance (i.e., multivariant technique to test whether the item characteristics are comparable across manifest groups) [25] across gender was carried out, respectively, following the methodological approach outlined by Milfont and Fisher [28]. This analysis aimed to determine whether the questionnaire’s factor structure was invariant across these variables. The null hypothesis of factor invariance should not be rejected in case of existing differences below 0.010 and 0.015 in CFI and RMSEA values, respectively, among the successive constrained models [29].

## 3. Results

### 3.1. Empirical Assessment of the Proposed Scale

**Figure 1:**
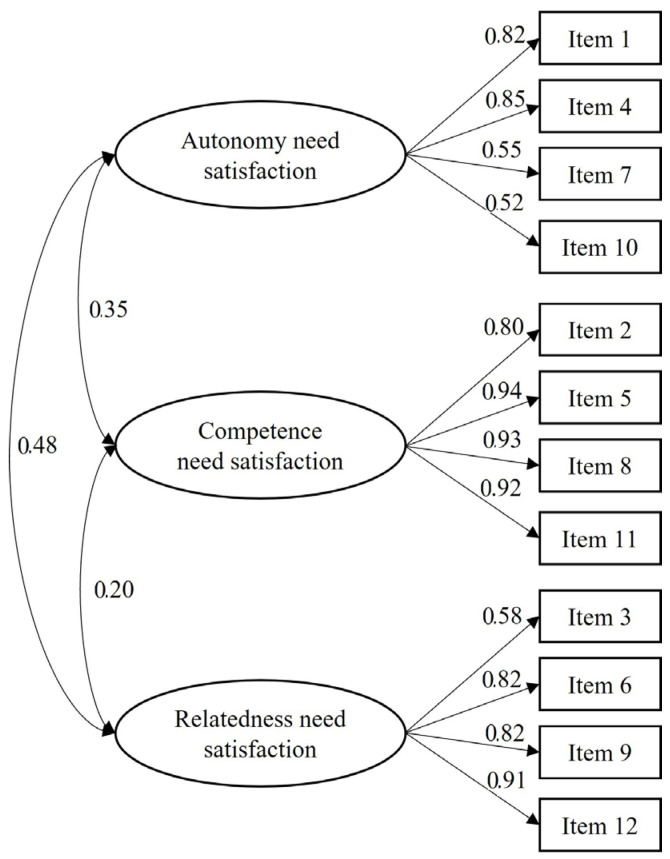
Confirmatory factor analysis.

The results of confirmatory factor analysis [χ^2^(51) = 220.51 (*p* ˂ 0.01); χ^2^/df = 4.32; CFI = 0.93; IFI = 0.93; TLI = 0.91; RMSEA = 0.10 (0.08–0.11)] indicated an acceptable fit to the data. Although the value of χ^2^/df and RMSEA exceeded the generally accepted range value, other models fit indices were adequate to the reference values. For example, CFI, IFI and TLI values were greater than 0.90, which is a criterion for good fit [14]. In addition, the standardised regression weights ranged from 0.52 to 0.94 (Figure 1).

As shown in Table 1, the internal consistency of the measures was accepted as the composite reliability values ranged from 0.78 (autonomy need satisfaction) to 0.94 (competence need satisfaction). The AVE values ranged from 0.50 (autonomy need satisfaction) to 0.81 (competence need satisfaction), indicating good convergent validity for all constructs. In addition, none of the squared correlations exceeded the AVE values for each associated construct. Thus, discriminant validity was accepted [27].

### 3.2. Multi-Group Factor Analysis of Invariance across Gender

Table 2 reflects the differences in CFI and RMSEA values below 0.010 and 0.015, respectively, in each of the successive restricted models for the six-factor correlated structure. The results indicate that the null hypothesis of factor invariance between gender cannot be rejected [29].

## 4. Discussion

This study performed a confirmatory factor analysis to translate the BPNS-ACS scale into Portuguese and evaluate its psychometric properties and validity. This analysis indicated an acceptable fit to the data, showing internal consistency, invariance across gender, and convergent and divergent validity. Therefore, the Portuguese version of the BPNS-ACS scale can be considered a valid measure to access autonomy, competence, and relatedness satisfaction in the ACS context among Portuguese youth.

In line with the initial validation of the BPNS-ACS scale in Spanish young people [21], the three-factor confirmatory factor analysis model presented an acceptable fit to the data, and all factors loadings were greater than 0.50 supporting the relationship of each item with its pre-defined factor.. Furthermore, the observed moderate association between the autonomy, competence and relatedness factors follows the theoretical distinction between these three BPN proposed by Ryan and Deci [10]. Additionally, previous research on ACS [21] and physical activity [16,17,18] have observed autonomy, competence, and relatedness need satisfaction to be positively related with these behaviours. These findings are important and reinforce the need for self-efficacy and experience mastery for ACS, as suggested by Burgueño, González-Cutre, Sevil-Serrano, Herrador-Colmenero, Segura-Díaz, Medina-Casaubón and Chillon [21].

Research based on the SDT suggests that young people who have their BPN satisfied are more committed and motivated to engage in physical activity [30]. Having the support of a comprehensive conceptual framework to understand health behaviours benefits the planning and implementation of health promoting interventions. In this sense, an understanding of the relationships between autonomy, competence and relatedness needs satisfaction and ACS behaviour is of great interest, as it could help in the development of appropriate strategies and approaches to promote physical activity in young people through active commuting. This is especially important for health promotion agents and institutions, enabling them to develop a conducive school environment for the promotion of physical activity [31,32].

Multi-group factor analysis sustained the measurement invariance across gender of the three-factor confirmatory factor analysis model for the Portuguese version of the BPNS-ACS scale, which agrees with previous research on the same questionnaire among Spanish youth [21]. The measurement of invariance demonstrates that boys and girls have a similar understanding of the questionnaire’s items. Thus, the BPNS-ACS scale can be applied to both genders.

The current study presents a set of limitations that should be acknowledged. This study employed a cross-sectional design, not allowing to ascertain the direction of causality of the associations tested. Thus, it is not possible to know whether BPN impacted ACS or if this behaviour impacts BPN. Additionally, the sampling procedures and Portugal’s specific cultural and social context impose caution in generalising the results beyond this scope. Because of this, cultural and linguistic adaptations of the BPNS-ACS scale for other contexts and the test of its psychometric properties should continue to be performed in future studies.

## 5. Conclusions

This study presented the translation and validation of the Portuguese version of the BPNS-ACS scale, adapted from the initial BPNS-ACS scale in the Spanish context [10]. The evaluation of psychometric properties provided evidence of the adequacy of this questionnaire among Portuguese youth aged 11 to 19 years old. Therefore, the Portuguese version of the BPNS-ACS scale constitutes a valid questionnaire used in the Portuguese context. The validation of the Portuguese version of the BPNS-ACS scale allows the application of this instrument in a Portuguese speaking context and the development of studies that help to better understand the factors that may regulate ACS behaviour in young people.

## Figures and Tables

**Table 1:** Means and standard deviations, factor loadings; construct reliability; average variance extracted; and correlations among constructs.

Construct/Items	M (SD)	L	CR	AVE
Autonomy need satisfaction			0.78	0.50
1. A forma como vou para a escola ajusta-se ao que quero. (Mi modo habitual de ir al colegio se ajusta a lo que yo quiero) (I feel that my usual mode of commuting to and from school fits well with what I want)	3.92 (1.20)	0.82		
2. A forma como vou para a escola corresponde à forma como gostaria de ir. (La forma de desplazarme al colegio coincide perfectamente con como yo quiero ir) (I feel that the mode of commuting to and from school coincides with how I want to travel)	3.71 (1.29)	0.85		
3. Vou para a escola como me apetece. (La forma de desplazarme al colegio es la que me apetece) (I feel that the mode of commuting to and from school is what I like)	3.10 (1.47)	0.55		
4. Posso escolher a forma como vou para a escola. (Puedo elegir cómo desplazarme al colégio) (I feel that I can choose how to commute to and from school)	3.03 (1.46)	0.52		
Competence need satisfaction			0.94	0.81
5. Sinto que consigo ir para a escola a pé ou de bicicleta. (Me siento capaz de ir andando o en bici al colegio) (I feel able to walk or cycle to and from school)	3.08 (1.62)	0.80		
6. Sinto que tenho as habilidades necessárias para ir a pé ou de bicicleta para a escola. (Tengo las habilidades necesarias para ir andando o en bici al colegio sin problemas) (I feel that I have the necessary skills to walk or cycle to and from school)	3.53 (1.49)	0.94		
7. Tenho habilidade para ir para a escola a pé ou de bicicleta. (Soy hábil para desplazarme al colegio andando o en bici) (I feel skilled to walk or cycle to and from school)	3.47 (1.54)	0.93		
8. Sinto-me capaz de deslocar-me a pé ou de bicicleta para a escola. (Me siento capacitado/a para desplazarme en bici o andando al colegio) (I feel competent to walk or cycle to and from school)	3.39 (1.56)	0.92		
Relatedness need satisfaction			0.87	0.63
9. Sinto-me confortável quando vou para a escola acompanhado. (Me siento muy cómodo/a cuando voy al colegio acompañado/a) (I feel extremely comfortable when someone accompanied me to school)	4.22 (1.00)	0.58		
10. Dou-me bem com as pessoas que me acompanham quando vou para a escola. (Me relaciono de forma muy amistosa con los/las que me acompañan) (I friendly interact with who accompanies me to school)	4.45 (0.95)	0.82		
11. Sinto que posso falar abertamente com as pessoas que me acompanham para a escola. (Siento que me puedo comunicar abiertamente con los/as que me acompañan) (I feel that I can openly communicate with who accompanies me to school)	4.02 (1.19)	0.82		
12. Sinto-me muito confortável com as pessoas que me acompanham para a escola. (Me siento muy cómodo/a con los/as que me acompañan) (I feel very comfortable with who accompanies me to school)	4.21 (1.13)	0.91		
	Correlation matrix			
Construct	1	2	3	
1. Autonomy need satisfaction	1.00			
2. Competence need satisfaction	0.12 *	1.00		
3. Relatedness need satisfaction	0.23 *	0.04 *	1.00	

Notes: No correlations failed the AVE test of discriminant validity; * *p* < 0.01. Abbreviations: AVE, average variance extracted; CR, construct reliability; M, mean; L, loadings; SD, standard deviations.

**Table 2:** Multi-group factor analysis of invariance.

	χ^2^	df	χ^2^/df	CFI	IFI	TLI	SRMR	RMSEA (90% CI)	MC	∆χ^2^	∆df	∆CFI	∆RMSEA
Configural invariance	311.13	102	3.05	0.92	0.92	0.90	0.06	0.08 (0.07 0.09)	--	--	--	--	--
Metric invariance	320.81	112	2.86	0.92	0.92	0.90	0.06	0.07 (0.06 0.08)	2 vs. 1	9.68	10	0.00	−0.01
Scalar invariance	328.18	121	2.71	0.92	0.92	0.91	0.06	0.07 (0.06 0.08)	3 vs. 2	7.37	9	0.00	0.00
Error variance invariance	381.72	133	2.87	0.91	0.91	0.90	0.06	0.07 (0.06 0.08)	4 vs. 3	54.53 *	12	0.01	0.00

Notes: * *p* < 0.01. Abbreviations: CFI, Comparative-of-Fit-Index; Df, degree of freedom; IFI, Bollen’s incremental fit index; MC, multiple comparisons; RMSEA, root mean square error of approximation; SRMR, standardized root mean squared residual; TLI, Tucker–Lewis index.

## Data Availability

The datasets generated and/or analysed during the current study are not publicly available due the terms of consent/assent to which the participants agreed, but are available from the corresponding author upon reasonable request. Please contact the corresponding author to discuss availability of data and materials.
